# (2*E*)-2-[(2*E*)-3-Phenyl­prop-2-en-1-yl­idene]-2,3-dihydro-1*H*-inden-1-one

**DOI:** 10.1107/S1600536812007131

**Published:** 2012-02-24

**Authors:** Abdullah M. Asiri, Hassan M. Faidallah, Khulud F. Al-Nemari, Seik Weng Ng, Edward R. T. Tiekink

**Affiliations:** a Chemistry Department, Faculty of Science, King Abdulaziz University, PO Box 80203, Jeddah, Saudi Arabia; b The Center of Excellence for Advanced Materials Research, King Abdulaziz University, Jeddah, PO Box 80203, Saudi Arabia; c Department of Chemistry, University of Malaya, 50603 Kuala Lumpur, Malaysia

## Abstract

The title indan-1-one derivative, C_18_H_14_O, is planar with an r.m.s. deviation for all 19 non-H atoms of 0.098 Å. The conformation about each of the C=C bonds [1.343 (3) and 1.349 (3) Å] is *E*. Supra­molecular layers in the *bc* plane, mediated by C—H⋯O and π–π [ring centroid–centroid distance = 3.5282 (15) Å] inter­actions, feature in the crystal packing.

## Related literature

For the activity of related species developed for the treatment of Chagas disease, see: Vera-DiVaio *et al.* (2009 ▶). For the crystal structure of a closely related compound, see: Magomedova *et al.* (1980 ▶).
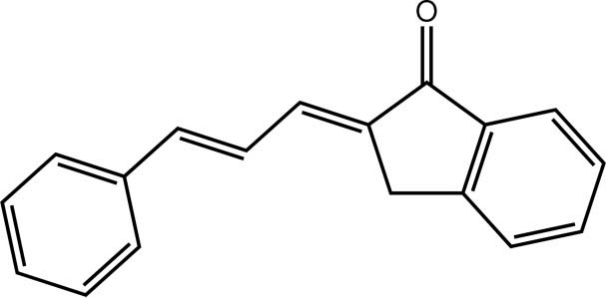


## Experimental

### 

#### Crystal data

C_18_H_14_O
*M* *_r_* = 246.29
Orthorhombic, 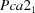

*a* = 29.192 (4) Å
*b* = 3.9110 (3) Å
*c* = 11.2025 (7) Å
*V* = 1279.0 (2) Å^3^
*Z* = 4
Mo *K*α radiation
μ = 0.08 mm^−1^
*T* = 100 K
0.25 × 0.15 × 0.10 mm

#### Data collection

Agilent SuperNova Dual diffractometer with an Atlas detector
Absorption correction: multi-scan (*CrysAlis PRO*; Agilent, 2011 ▶) *T*_min_ = 0.981, *T*_max_ = 0.992
3652 measured reflections
1521 independent reflections
1375 reflections with *I* > 2σ(*I*)
*R*_int_ = 0.035

#### Refinement

*R*[*F* ^2^ > 2σ(*F* ^2^)] = 0.042
*wR*(*F* ^2^) = 0.101
*S* = 1.05
1521 reflections
172 parameters
1 restraint
H-atom parameters constrained
Δρ_max_ = 0.19 e Å^−3^
Δρ_min_ = −0.20 e Å^−3^

### 

Data collection: *CrysAlis PRO* (Agilent, 2011 ▶); cell refinement: *CrysAlis PRO*; data reduction: *CrysAlis PRO*; program(s) used to solve structure: *SHELXS97* (Sheldrick, 2008 ▶); program(s) used to refine structure: *SHELXL97* (Sheldrick, 2008 ▶); molecular graphics: *ORTEP-3* (Farrugia, 1997 ▶) and *DIAMOND* (Brandenburg, 2006 ▶); software used to prepare material for publication: *publCIF* (Westrip, 2010 ▶).

## Supplementary Material

Crystal structure: contains datablock(s) global, I. DOI: 10.1107/S1600536812007131/pv2516sup1.cif

Structure factors: contains datablock(s) I. DOI: 10.1107/S1600536812007131/pv2516Isup2.hkl

Supplementary material file. DOI: 10.1107/S1600536812007131/pv2516Isup3.cml

Additional supplementary materials:  crystallographic information; 3D view; checkCIF report

## Figures and Tables

**Table 1:** Hydrogen-bond geometry (Å, °)

*D*—H⋯*A*	*D*—H	H⋯*A*	*D*⋯*A*	*D*—H⋯*A*
C8—H8*A*⋯O1^i^	0.99	2.58	3.432 (3)	144

Symmetry code: (i) 
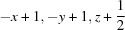
.

**Fig. 1.**
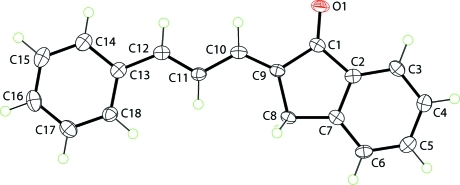
The molecular structure of (I) showing the atom-labelling scheme and displacement ellipsoids at the 50% probability level.

**Fig. 2.**
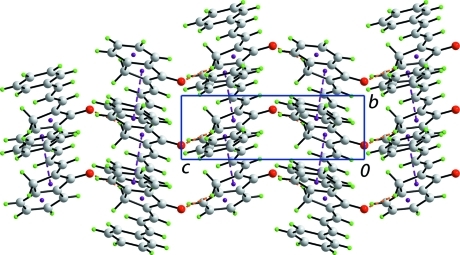
A view of the supramolecular layer in the bc plane in (I). The C—H···O and π–π interactions are shown as orange and purple dashed lines, respectively.

**Fig. 3.**
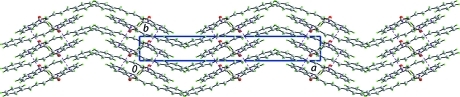
A view in projection down the c axis of the unit-cell contents of (I). The C—H···O and π–π interactions are shown as orange and purple dashed lines, respectively.

## supplementary crystallographic information

### Comment

The title compound (I), was investigated owing to its relationship to some active compounds developed for the treatment of Chagas disease (Vera-DiVaio *et al.*, 2009).

The molecule of (I) (Fig. 1), is planar with a r.m.s. deviation for all 19 non-hydrogen atoms = 0.098 Å. The maximum deviations are found for the C12 [0.138 (2) Å] and C15 [-0.154 (3) Å] atoms. The configuration about the C9═C10 bond [1.343 (3) Å] is *E* and a similar configuration is found for the C11═C12 bond [1.349 (3) Å].

In the crystal packing, C—H···O (Table 1), and π–π [ring centroid(C1,C2,C7–C9)···centroid(C2–C7)^i^ distance = 3.5282 (15) Å, angle between planes = 1.49 (13)°, for symmetry operation *i*: *x*, 1 + *y*, *z*] interactions link molecules into layers in the *bc* plane (Fig. 2). The layers stack along the *a* axis with no specific interactions between them (Fig. 2).

### Experimental

A solution of cinnamaldehyde (1.3 g, 0.01 mol) in ethanol (20 ml) was added to a stirred solution of 1-indanone (1.3 g, 0.01 mol) in ethanolic KOH (20%, 20 ml), and stirring was maintained at room temperature for 6 h. The reaction mixture was then poured onto water (200 ml) and set aside overnight. The precipitated product was collected by filtration, washed with water, dried and recrystallized from its ethanol solution as prisms, m.p.: 390–391 K.

### Refinement

Carbon-bound H-atoms were placed in calculated positions [C—H = 0.95 to 0.99 Å, *U*_iso_(H) = 1.2*U*_eq_(C)] and were included in the refinement in the riding model approximation. In the absence of significant anomalous scattering effects, 521 Friedel pairs were averaged in the final refinement.

### Crystal data

**Table:**

C_18_H_14_O	*F*(000) = 520
*M**_r_* = 246.29	*D*_x_ = 1.279 Mg m^−^^3^
Orthorhombic, *P**c**a*2_1_	Mo *K*α radiation, λ = 0.71073 Å
Hall symbol: P 2c -2ac	Cell parameters from 1378 reflections
*a* = 29.192 (4) Å	θ = 2.8–27.5°
*b* = 3.9110 (3) Å	µ = 0.08 mm^−^^1^
*c* = 11.2025 (7) Å	*T* = 100 K
*V* = 1279.0 (2) Å^3^	Prism, light-brown
*Z* = 4	0.25 × 0.15 × 0.10 mm

### Data collection

**Table:**

Agilent SuperNova Dual diffractometer with an Atlas detector	1521 independent reflections
Radiation source: SuperNova (Mo) X-ray Source	1375 reflections with *I* > 2σ(*I*)
Mirror monochromator	*R*_int_ = 0.035
Detector resolution: 10.4041 pixels mm^-1^	θ_max_ = 27.6°, θ_min_ = 2.8°
ω scan	*h* = −21→38
Absorption correction: multi-scan (*CrysAlis PRO*; Agilent, 2011)	*k* = −3→4
*T*_min_ = 0.981, *T*_max_ = 0.992	*l* = −14→9
3652 measured reflections	

### Refinement

**Table:**

Refinement on *F*^2^	Primary atom site location: structure-invariant direct methods
Least-squares matrix: full	Secondary atom site location: difference Fourier map
*R*[*F*^2^ > 2σ(*F*^2^)] = 0.042	Hydrogen site location: inferred from neighbouring sites
*wR*(*F*^2^) = 0.101	H-atom parameters constrained
*S* = 1.05	*w* = 1/[σ^2^(*F*_o_^2^) + (0.0502*P*)^2^ + 0.2004*P*] where *P* = (*F*_o_^2^ + 2*F*_c_^2^)/3
1521 reflections	(Δ/σ)_max_ = 0.001
172 parameters	Δρ_max_ = 0.19 e Å^−^^3^
1 restraint	Δρ_min_ = −0.20 e Å^−^^3^

### Fractional atomic coordinates and isotropic or equivalent isotropic displacement parameters (Å^2^)

**Table:**

	*x*	*y*	*z*	*U*_iso_*/*U*_eq_	
O1	0.44657 (6)	0.7797 (5)	0.50084 (17)	0.0279 (4)	
C1	0.44960 (8)	0.6972 (6)	0.6062 (2)	0.0198 (5)	
C2	0.41457 (9)	0.5162 (6)	0.6782 (2)	0.0199 (5)	
C3	0.37141 (9)	0.3984 (6)	0.6439 (2)	0.0228 (5)	
H3	0.3609	0.4261	0.5642	0.027*	
C4	0.34418 (9)	0.2399 (6)	0.7290 (3)	0.0250 (6)	
H4	0.3149	0.1542	0.7073	0.030*	
C5	0.35962 (9)	0.2057 (6)	0.8465 (2)	0.0250 (6)	
H5	0.3405	0.1003	0.9043	0.030*	
C6	0.40277 (9)	0.3243 (6)	0.8799 (2)	0.0226 (5)	
H6	0.4130	0.3005	0.9600	0.027*	
C7	0.43058 (8)	0.4770 (6)	0.7953 (2)	0.0192 (5)	
C8	0.47848 (8)	0.6172 (6)	0.8098 (2)	0.0194 (5)	
H8A	0.5003	0.4344	0.8323	0.023*	
H8B	0.4794	0.7996	0.8710	0.023*	
C9	0.48938 (9)	0.7589 (6)	0.6868 (2)	0.0184 (5)	
C10	0.52758 (8)	0.9192 (6)	0.6502 (2)	0.0205 (5)	
H10	0.5276	1.0052	0.5709	0.025*	
C11	0.56849 (8)	0.9734 (6)	0.7194 (2)	0.0199 (5)	
H11	0.5694	0.8957	0.7997	0.024*	
C12	0.60551 (9)	1.1316 (6)	0.6731 (2)	0.0222 (5)	
H12	0.6023	1.2158	0.5940	0.027*	
C13	0.65002 (9)	1.1892 (6)	0.7300 (2)	0.0220 (5)	
C14	0.68584 (9)	1.3265 (6)	0.6620 (3)	0.0279 (6)	
H14	0.6805	1.3881	0.5811	0.033*	
C15	0.72899 (9)	1.3738 (7)	0.7109 (3)	0.0310 (6)	
H15	0.7530	1.4639	0.6630	0.037*	
C16	0.73741 (9)	1.2911 (7)	0.8289 (3)	0.0317 (7)	
H16	0.7669	1.3262	0.8625	0.038*	
C17	0.70216 (9)	1.1559 (7)	0.8976 (3)	0.0290 (6)	
H17	0.7078	1.0967	0.9785	0.035*	
C18	0.65895 (9)	1.1062 (7)	0.8501 (2)	0.0234 (6)	
H18	0.6352	1.0157	0.8986	0.028*	

### Atomic displacement parameters (Å^2^)

**Table:**

	*U*^11^	*U*^22^	*U*^33^	*U*^12^	*U*^13^	*U*^23^
O1	0.0310 (10)	0.0385 (11)	0.0142 (8)	0.0023 (8)	−0.0007 (8)	0.0023 (8)
C1	0.0217 (12)	0.0194 (11)	0.0183 (11)	0.0062 (9)	−0.0006 (10)	−0.0037 (11)
C2	0.0244 (12)	0.0157 (11)	0.0197 (11)	0.0054 (10)	0.0024 (10)	−0.0018 (10)
C3	0.0241 (13)	0.0201 (11)	0.0242 (12)	0.0047 (10)	−0.0054 (11)	−0.0020 (11)
C4	0.0215 (12)	0.0218 (13)	0.0317 (15)	0.0015 (10)	0.0014 (12)	−0.0006 (12)
C5	0.0255 (14)	0.0203 (12)	0.0292 (14)	0.0031 (10)	0.0056 (11)	0.0025 (11)
C6	0.0286 (14)	0.0197 (12)	0.0194 (12)	0.0044 (10)	0.0019 (11)	0.0023 (11)
C7	0.0225 (12)	0.0147 (11)	0.0205 (11)	0.0063 (9)	−0.0015 (10)	−0.0039 (10)
C8	0.0231 (13)	0.0204 (12)	0.0147 (11)	0.0025 (9)	−0.0015 (10)	−0.0022 (10)
C9	0.0218 (12)	0.0182 (11)	0.0153 (11)	0.0039 (9)	0.0000 (9)	−0.0014 (10)
C10	0.0267 (12)	0.0181 (11)	0.0166 (10)	0.0048 (9)	0.0014 (10)	0.0001 (10)
C11	0.0224 (13)	0.0209 (12)	0.0164 (11)	0.0026 (10)	0.0015 (10)	−0.0001 (11)
C12	0.0280 (13)	0.0185 (12)	0.0203 (12)	0.0018 (9)	0.0008 (11)	−0.0007 (11)
C13	0.0261 (13)	0.0163 (12)	0.0235 (13)	0.0004 (9)	0.0033 (12)	−0.0048 (11)
C14	0.0297 (14)	0.0248 (13)	0.0292 (14)	−0.0030 (10)	0.0052 (12)	−0.0043 (12)
C15	0.0265 (14)	0.0284 (14)	0.0382 (16)	−0.0047 (11)	0.0089 (13)	−0.0055 (13)
C16	0.0236 (14)	0.0299 (14)	0.0417 (17)	−0.0004 (11)	−0.0039 (13)	−0.0105 (14)
C17	0.0307 (15)	0.0295 (13)	0.0268 (14)	0.0020 (12)	−0.0056 (12)	−0.0048 (12)
C18	0.0240 (13)	0.0247 (13)	0.0215 (12)	−0.0007 (10)	0.0024 (11)	−0.0015 (11)

### Geometric parameters (Å, º)

**Table:**

O1—C1	1.227 (3)	C10—C11	1.439 (3)
C1—C2	1.482 (3)	C10—H10	0.9500
C1—C9	1.491 (3)	C11—C12	1.349 (3)
C2—C3	1.396 (3)	C11—H11	0.9500
C2—C7	1.401 (3)	C12—C13	1.465 (3)
C3—C4	1.388 (4)	C12—H12	0.9500
C3—H3	0.9500	C13—C14	1.401 (4)
C4—C5	1.398 (4)	C13—C18	1.408 (4)
C4—H4	0.9500	C14—C15	1.386 (4)
C5—C6	1.393 (4)	C14—H14	0.9500
C5—H5	0.9500	C15—C16	1.382 (4)
C6—C7	1.383 (4)	C15—H15	0.9500
C6—H6	0.9500	C16—C17	1.390 (4)
C7—C8	1.511 (3)	C16—H16	0.9500
C8—C9	1.518 (3)	C17—C18	1.383 (4)
C8—H8A	0.9900	C17—H17	0.9500
C8—H8B	0.9900	C18—H18	0.9500
C9—C10	1.343 (3)		
			
O1—C1—C2	126.8 (2)	C1—C9—C8	109.1 (2)
O1—C1—C9	126.6 (2)	C9—C10—C11	126.4 (2)
C2—C1—C9	106.6 (2)	C9—C10—H10	116.8
C3—C2—C7	121.5 (2)	C11—C10—H10	116.8
C3—C2—C1	129.1 (2)	C12—C11—C10	121.7 (2)
C7—C2—C1	109.4 (2)	C12—C11—H11	119.2
C4—C3—C2	118.4 (3)	C10—C11—H11	119.2
C4—C3—H3	120.8	C11—C12—C13	127.9 (2)
C2—C3—H3	120.8	C11—C12—H12	116.1
C3—C4—C5	120.3 (3)	C13—C12—H12	116.1
C3—C4—H4	119.8	C14—C13—C18	118.0 (2)
C5—C4—H4	119.8	C14—C13—C12	118.9 (2)
C6—C5—C4	120.8 (2)	C18—C13—C12	123.0 (2)
C6—C5—H5	119.6	C15—C14—C13	120.9 (3)
C4—C5—H5	119.6	C15—C14—H14	119.5
C7—C6—C5	119.4 (2)	C13—C14—H14	119.5
C7—C6—H6	120.3	C16—C15—C14	120.6 (3)
C5—C6—H6	120.3	C16—C15—H15	119.7
C6—C7—C2	119.5 (2)	C14—C15—H15	119.7
C6—C7—C8	128.8 (2)	C15—C16—C17	119.2 (3)
C2—C7—C8	111.7 (2)	C15—C16—H16	120.4
C7—C8—C9	103.25 (19)	C17—C16—H16	120.4
C7—C8—H8A	111.1	C18—C17—C16	121.0 (3)
C9—C8—H8A	111.1	C18—C17—H17	119.5
C7—C8—H8B	111.1	C16—C17—H17	119.5
C9—C8—H8B	111.1	C17—C18—C13	120.3 (2)
H8A—C8—H8B	109.1	C17—C18—H18	119.8
C10—C9—C1	122.5 (2)	C13—C18—H18	119.8
C10—C9—C8	128.4 (2)		
			
O1—C1—C2—C3	0.5 (4)	O1—C1—C9—C8	179.6 (2)
C9—C1—C2—C3	−179.3 (2)	C2—C1—C9—C8	−0.5 (3)
O1—C1—C2—C7	−178.5 (2)	C7—C8—C9—C10	178.8 (2)
C9—C1—C2—C7	1.6 (3)	C7—C8—C9—C1	−0.7 (2)
C7—C2—C3—C4	0.1 (4)	C1—C9—C10—C11	−176.3 (2)
C1—C2—C3—C4	−178.8 (2)	C8—C9—C10—C11	4.3 (4)
C2—C3—C4—C5	1.1 (4)	C9—C10—C11—C12	178.5 (2)
C3—C4—C5—C6	−1.1 (4)	C10—C11—C12—C13	−176.6 (2)
C4—C5—C6—C7	−0.1 (4)	C11—C12—C13—C14	173.1 (2)
C5—C6—C7—C2	1.3 (3)	C11—C12—C13—C18	−5.5 (4)
C5—C6—C7—C8	−178.7 (2)	C18—C13—C14—C15	1.1 (4)
C3—C2—C7—C6	−1.3 (3)	C12—C13—C14—C15	−177.6 (2)
C1—C2—C7—C6	177.8 (2)	C13—C14—C15—C16	−1.0 (4)
C3—C2—C7—C8	178.7 (2)	C14—C15—C16—C17	0.7 (4)
C1—C2—C7—C8	−2.2 (3)	C15—C16—C17—C18	−0.6 (4)
C6—C7—C8—C9	−178.2 (2)	C16—C17—C18—C13	0.6 (4)
C2—C7—C8—C9	1.8 (2)	C14—C13—C18—C17	−0.9 (4)
O1—C1—C9—C10	0.1 (4)	C12—C13—C18—C17	177.8 (2)
C2—C1—C9—C10	180.0 (2)		

### Hydrogen-bond geometry (Å, º)

**Table:**

*D*—H···*A*	*D*—H	H···*A*	*D*···*A*	*D*—H···*A*
C8—H8*A*···O1^i^	0.99	2.58	3.432 (3)	144

Symmetry code: (i) −*x*+1, −*y*+1, *z*+1/2.
